# Reviewed article: Pimenta IT, Marsico EFC, Souto APM, Geraldo MCHM, Amaral LF, Duffrayer KM, Ribeiro CLP, Aguilar GMO. Vigilância em saúde em eventos de massa: relato da experiência do Centro de Informações Estratégicas em Vigilância em Saúde da cidade do Rio de Janeiro, 2024. Epidemiol Serv Saude. 2025:34;e20250212

**DOI:** 10.1590/S2237-96222025v34e20250212.b

**Published:** 2025-09-29

**Authors:** Antônio Ygor Modesto de Oliveira

**Affiliations:** 1 Ministério da Saúde, Brasília, DF, Brasil

## Peer review B

## Questionnaire

Does the manuscript contain new and significant information to justify publication? Yes

Does the Abstract (Summary) clearly and accurately describe the content of the article? Yes

Is the problem significant and concisely stated? Yes

Are the methods described comprehensively? Yes

Are the interpretations and conclusions justified by the results? Yes

Is adequate reference made to other work in the field? Yes

## Manuscript Structure

Length of article is: Adequate

Number of tables is: Adequate

Number of figures is: Adequate

Please state any conflict(s) of interest that you have in relation to the review of this paper. None

Interest: Excellent

Quality: Excellent

Originality: Excellent 

Overall: Excellent

Recommendation: Accept

## Comments

Prezado(s) autor(es),

Seu relato de experiência apresenta de forma clara e concisa uma experiência de vigilância em saúde para eventos de massa, permitindo que a estratégia adotada possa ser replicada e implementada por outros Centros de Informações Estratégicas em Vigilância em Saúde, contribuindo para o fortalecimento da Vigilância em Saúde e Ambiente e consequentemente do Sistema Único de Saúde.

O estudo descreve o método de forma clara e objetiva, informando as ações realizadas em cada etapa da estratégia adotada para a vigilância do evento, e aborda de forma concisa os resultados obtidos por essa estratégia. Considero que não é necessário realizar ajustes no manuscrito.

